# BAK and BAX: Therapeutic Targets for Acute Myocardial Infarction and Myocardial Ischemia-Reperfusion Injury

**DOI:** 10.3390/biology15010081

**Published:** 2025-12-31

**Authors:** Zejun Xu, Fei Meng, Hongjun Yang, Yaling Liu, Kaiqin Ye, Fei Qin, Dongyan Liu, Haiming Dai

**Affiliations:** 1 Key Laboratory of Key Technology Research on Chemical Raw Materials and Preparations of Guangdong Province, Department of Science and Technology of Guangdong Province, People’s Government of Guangdong Province, Guangzhou 510515, China; 2 Institute of Pharmacology, GuangZhou BaiYunShan Pharmaceutical Holdings Co., Ltd., BaiYunShan Pharmaceutical General Factory, Guangzhou 510410, China; 3 College of Life Sciences, University of Science and Technology of China, Hefei 230026, China; tonymeng@ustc.edu.cn (F.M.);; 4 Beijing Key Laboratory of Traditional Chinese Medicine Basic Research on Prevention and Treatment for Major Diseases, Experimental Research Center, China Academy of Chinese Medical Sciences, Beijing 100700, China; 5 School of Life Science and Engineering, Southwest University of Science and Technology, Mianyang 621000, China; 6 Anhui Province Key Laboratory of Medical Physics and Technology, Institute of Health and Medical Technology, Hefei Institutes of Physical Science, Chinese Academy of Sciences, Hefei 230031, China; kaiqinye@cmpt.ac.cn; 7 Hefei Cancer Hospital, Chinese Academy of Sciences, Hefei 230031, China; 8 Department of Molecular Pharmacology and Experimental Therapeutics, Mayo Clinic, Rochester, MN 55905, USA

**Keywords:** BCL2 family, acute myocardial infarction, MOMP, MPTP, apoptosis, necroptosis

## Abstract

Acute myocardial infarction (AMI) is a significant factor leading to the death of patients with coronary heart disease. Both AMI and reperfusion therapy after AMI cause myocardial cell death, which plays a significant role in heart failure. Following the restoration of blood flow during reperfusion, myocardial cells generate a large amount of oxygen free radicals, causing various forms of myocardial ischemia–reperfusion (IR) injury (IRI), ultimately leading to multiple types of myocardial cell death, among which apoptosis and necroptosis are the two major types. Given the extremely limited regenerative capacity of myocardium, inhibiting myocardial cell apoptosis and necroptosis is a key strategy for reducing mortality in patients with AMI. Both apoptosis and necroptosis are regulated by the BCL2 family of proteins, which were modulated by multiple signaling pathways, converging at BAK/BAX-mediated mitochondrial outer membrane permeabilization (MOMP), as well as mitochondrial inner membrane permeabilization (MIMP). BAK/BAX double knock out (DKO) mice showed reduced cell apoptosis, necroptosis, and infarct size in AMI animal models compared to wild type. This review describes the role of BCL2 family proteins in regulating apoptotic and necroptotic myocardial cell death during AMI and IR, explores the upstream pathways modulating apoptosis and necroptosis, and summarizes the recent advances in targeting BAK and/or BAX for cardiac protection. In addition, targeted delivery of BAK/BAX inhibitors to cardiomyocytes during AMI or myocardial IR has the potential to reduce myocardial cell death and therefore lower the mortality and enhance long-term prognosis for myocardial infarction patients.

## Simple Summary

A heart attack occurs when blood flow to the heart is blocked, causing the death of precious heart muscle cells. This not only damages the heart during the attack itself but can continue even after blood flow is restored, a phenomenon known as reperfusion injury. A major reason for this ongoing damage is the self-destruction of heart cells through processes called apoptosis and necroptosis. Our review focuses on two key proteins inside these cells, BAK and BAX, which act as central switches to initiate such cell death. We explain how various signals during a heart attack converge to activate BAK and BAX, leading to irreversible damage. Importantly, studies show that genetically removing BAK and BAX can significantly protect heart cells and reduce injury in animal models. We also summarize the exciting progress in developing drugs that can inhibit BAK and BAX. By targeting these key proteins, we hope to create new therapies that shield the heart during a heart attack, preserve its function, and ultimately save lives by limiting the loss of heart muscle cells.

## 1. Introduction

Focusing on apoptosis and mitochondrial-permeability transition pore (MPTP)-driven necrosis in myocardial infarction, this review highlights the therapeutic potential of inhibiting the core effectors BAK and BAX to limit cell death and improve outcomes. AMI is still significant, with about 10–20% of patients experiencing an AMI death within the first year and about a 5% annual death rate in the long term [1,2,3]. Although the importance of cell death depends on the disease, studies have indicated that cardiomyocyte cell death during AMI and IR following infarction plays an important role in cardiac dysfunction and heart failure [4,5,6,7,8]. Multiple cell death pathways [9] are involved after myocardial infarction, including the death receptor- and mitochondrial-mediated apoptotic pathways [8,10,11], the MPTP-dependent cell death pathway [12,13], the necroptosis pathway [13], the pyroptosis pathway [14], and the ferroptosis pathway [15,16]. Quite a few recent studies not only identified F_1_-F_0_ ATP synthase as a component of MPTP complex [17,18,19,20] but also developed several small molecules to inhibit apoptosis and MPTP-dependent cell death [21,22,23,24,25]. Therefore, in this review, we will focus on the AMI- and IR-induced apoptosis and necroptosis pathways. We will first revisit the apoptosis and MPTP-dependent cell death pathways, then summarize the recent advances in targeting these two pathways, in particular the two essential molecules BAK and BAX, to inhibit AMI- and IR-induced cell death. As a narrative review, this article aims to summarize and evaluate recent advances in the regulation of myocardial cell death by BCL2 family proteins during AMI and myocardial IR, particularly focusing on the two executive BCL2 family proteins BAK and BAX.

## 2. Apoptosis and MPTP-Driven Cell Death

Programmed cell death plays an important role in development, tissue homeostasis, and the initiation of the immune response. Programmed cell death can happen through different pathways, among which the apoptotic pathway is one of the most dominant [26,27,28].

**Figure 1:**
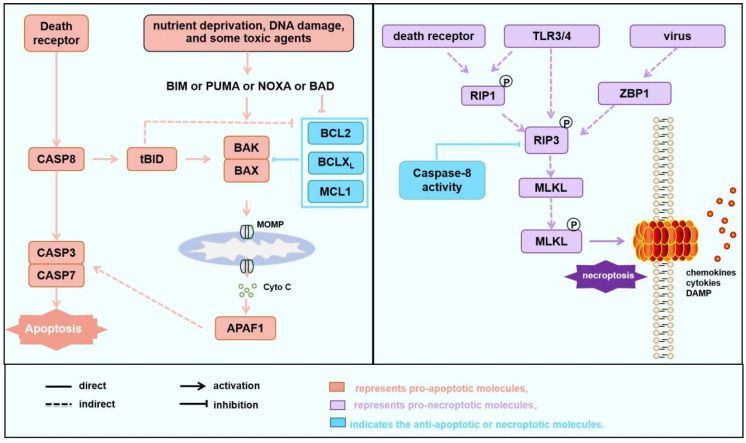
Cell death pathways involved in AMI and IR. Apoptosis and necroptosis pathways. Apoptosis (**left**) can be initiated through the death receptor pathway or the mitochondrial pathway. In the death receptor pathway, upon activation, death receptors directly activate downstream of the Caspase 8-Caspase 3/7 cascade, leading to the induction of apoptosis. Additionally, Caspase 8 cleaves BID into tBID, which subsequently activates BAK or BAX, amplifying the apoptosis through the mitochondrial apoptotic pathway. The mitochondrial apoptotic pathway can be activated under the influence of various factors such as nutrition deprivation, DNA damage, the induction of apoptosis-sensitizing proteins like PUMA, or the inhibition of anti-apoptotic proteins such as BCL2 by toxic agents, which mediates the oligomerization of BAK/BAX to mitochondrial outer membrane permeabilization (MOMP) and the release of cytochrome C, ultimately initiating apoptosis. Necroptosis (**right**): In cases where death receptor signaling is activated but apoptosis is insufficient or in the presence of viral infection or TLR activation, RIP3/RIP1 forms a phosphorylated complex (necrosome), which induces the phosphorylation of MLKL, which translocates to cell membrane, and induces necroptosis. In some cases, phosphorylated MLKL anchors to mitochondria, where it promotes the degradation of MCL1, liberating a small amount of free BAK or BAX. This stimulates the opening of the mitochondrial permeability transition pore (MPTP), leading to the release of mitochondrial matrix contents and the induction of necroptosis. Some studies have indicated that during the MPTP process, mitochondria release mtDNA to induce the cGAS/cGAMP/STING/TBK1 pathway, subsequently triggering inflammation through the NF-κB and IRF3 pathways [50,51,55,56].

Apoptotic cell death (apoptosis) can be performed either through the mitochondrial apoptotic pathway, which is regulated by BCL2 family proteins, or through the extrinsic death receptor pathway, which is initiated after the death ligands bind to death receptors [29,30] (Figure 1, left). The mitochondrial apoptotic pathway is usually initiated after mitochondrial outer membrane permeabilization (MOMP), which is mostly and directly caused by the activation of BAK and/or BAX, the two executive pro-apoptotic BCL2 family members. Besides the two executive pro-apoptotic members, BCL2 family proteins also include pro-apoptotic BH3-only members (such as BIM, BID, and PUMA) and anti-apoptotic members (such as BCL2, BCLX_L_, and MCL1), controlling MOMP through their interactions. Upon apoptotic signaling, the balance between BCL2 family proteins is disrupted, and BAK or BAX will oligomerize at the mitochondrial outer membrane and permeabilize it, leading to the release of cytochrome C into the cytoplasm, followed by the activation of caspase-3 and caspase-9, and ultimately apoptosis [31,32].

In contrast to MOMP, MIMP is controlled by MPTP, which also refers to permeability transition pore complex (PTPC) [33,34]. MPTP serves as a non-selective channel, with Cyclophilin D (CypD) as the first identified regulator. The constituent proteins of MPTP also encompass adenine nucleotide translocase (ANT) and voltage-dependent anion channel (VDAC), among others. Factors such as calcium overload and oxidative stress can stimulate the opening of MPTP on the mitochondrial inner membrane [35]. Notably, recent studies have identified the F_1_-F_0_ ATP synthase as a structural component of the MPTP [18,19,20,36]. F_1_-F_0_ ATP synthase is one of the five key complexes in the mitochondrial respiratory chain, utilizing the energy of the proton gradient to synthesize ATP, thereby providing energy for cellular activities. The c-subunit of FO of the F_1_-F_0_ ATP synthase forms a voltage-sensitive channel, playing an essential role in MPTP opening under certain stimulations. The induction of MPTP opening necessitates the activation of CypD/the F_1_-F_0_ ATP synthase complex, leading to MIMP, which permits the free passage of substances with a molecular weight of less than 1.5 kD through the mitochondrial inner membrane [17]. Therefore, MPTP opening can be induced through two different mechanisms: the high- and low-conductance states [17,37,38,39]. A high-conductance state of mPTP open is induced by extremely stressful situations, which often happens through the F_1_-F_0_ ATP synthase. In contrast, a low-conductance state often occurs during the normal physiological activity of mitochondria, such as physiological Ca^2+^ accumulation inside the mitochondria, triggering the opening through ANT. The extreme calcium overload might also induce a high-conductance state of MPTP opening. Whether and how these two different mechanisms link together is still not clear.

MPTP opening primarily induces necrotic types of cell death but may also amplify apoptosis under certain conditions [37,38,39]. Necrotic types of cell death, often occurring in response to chemical or physical stress, is characterized by cell rupture, lysis, and release of cellular components to the extracellular environment, which initiates inflammation [28]. Compared to apoptosis, which eliminates a large number of cells without causing inflammation or causes minor inflammation, necrotic cell death induces inflammatory responses. Recent studies have identified that both MPTP and BAK/BAX play an important role in induction of necroptosis [40,41,42,43], a regulated form of necrotic type cell death, which is classically mediated by the RIPK1/PRIK3/MLKL pathway (Figure 1, right panel). Under certain conditions, when apoptosis fails to occur, for example, when caspase-8 is absent or inhibited, necroptosis serves as an alternative way for cell death [27]. Upon upstream signaling through death receptors such as tumor necrosis factor receptor (TNFR) and Toll-like receptor (TLR) family members, interferons, the initiation of necroptosis is triggered by autophosphorylation of RIPK1 at Ser166, a key event for its conformational activation [44]. Activated RIPK1 then interacts via its C-terminal RHIM domain with the homologous domain in RIPK3, leading to the formation of the core signaling protein complex known as the “necrosome” [45]. Within this complex, RIPK1 directly phosphorylates RIPK3 at Ser227, fully activating its kinase function [46]. Alternatively, RIPK3 is activated by intracellular RNA and DNA sensors such as ZBP1 (also named DAI) that are activated by certain viruses [47]. Subsequently, active RIPK3 recruits MLKL and phosphorylates MLKL at Thr357 and Ser358 within its pseudokinase domain, relieving autoinhibition [48]. This phosphorylation induces a conformational change in MLKL, exposing its N-terminal four-helix bundle, which will then oligomerize and translocate to the plasma membrane, where it forms transmembrane pores that disrupt membrane integrity, ultimately leading to lytic cell death [17]. Interestingly, other mechanisms regulating necroptosis have also been reported, which crosstalk between necroptosis and the mitochondrial apoptotic pathway. Some exposed MLKL pore complexes anchor to the mitochondrial outer membrane, inducing MCL1 degradation and minimal BAK/BAX activation, which in turn induce the opening of pores formed by the F_1_-F_0_ ATP synthase and CypD, releasing contents and triggering MPTP-driven necroptosis. The persistent opening of the MPTP will lead to the loss of integrity of mitochondria or plasma membranes, resulting in the release of cytochrome C and mtDNA, decreased ATP levels, and energy depletion. In addition to necroptosis, these conditions can also initiate other types of cell response or cell death, including apoptosis [49,50], pyroptosis [51] and mitophagy [52,53,54].

## 3. Protection of AMI- or IR-Related Mortality by Inhibitions of Myocardial Cell Apoptosis and/or Necroptosis

### 3.1. Rapid Calcium Overload and/or ROS Are Two Major Components Causing Myocardial Cell Death During AMI and IR

Apoptosis and necroptosis remain the primary modes of cell death during AMI and IR [54]. MI is caused by thrombotic obstruction of arteries or bypass grafts, which leads to a sudden decrease in myocardial blood flow and subsequent myocardial cell death due to hypoxia [57,58]. Clinical treatments for AMI include thrombolysis, percutaneous coronary intervention (PCI), and coronary artery bypass graft surgery [58]. Although improvements in coronary reperfusion intervention methods have significantly reduced mortality rates in patients [58], the incidence and mortality rates of myocardial functional damage, heart failure, and ischemia–reperfusion injury caused by acute myocardial infarction remain high [59,60,61]. The cardiovascular mortality rate within one year is about 2–6% in developed countries but approximately 11% worldwide by coronary artery reperfusion strategies [62]. The restoration of myocardial blood flow is crucial in preventing cell death following ischemia. Nevertheless, the reperfusion process of blood can further exacerbate myocardial cell death, leading to a series of complications such as reperfusion arrhythmia, myocardial stunning, and heart failure. IR is intricately linked to diverse biological processes, such as apoptosis, oxidative stress, inflammation, and ferroptosis [63]. Among these processes, apoptosis stands as the pivotal stage that holds a vital significance in IR. Given the extremely limited regenerative capacity of the myocardium, it is an important strategy for myocardial protection to explore more innovative and effective approaches to reduce cell death during myocardial ischemia and its subsequent processes.

**Figure 2:**
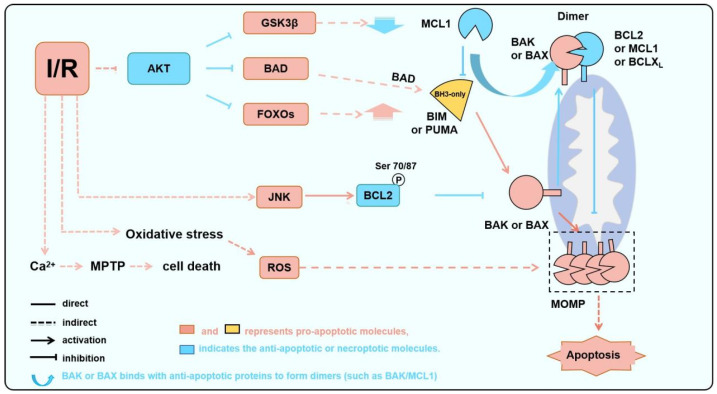
BAK/BAX serves as a critical node in IR-induced apoptosis. During IR, AKT is inhibited, JNK is abnormally activated, and ROS is increased due to oxidative stress. These processes lead to a decrease in anti-apoptotic protein levels or activities and an increase in pro-apoptotic protein levels or activities, thereby activating BAK or BAX and inducing apoptosis.

**Figure 3:**
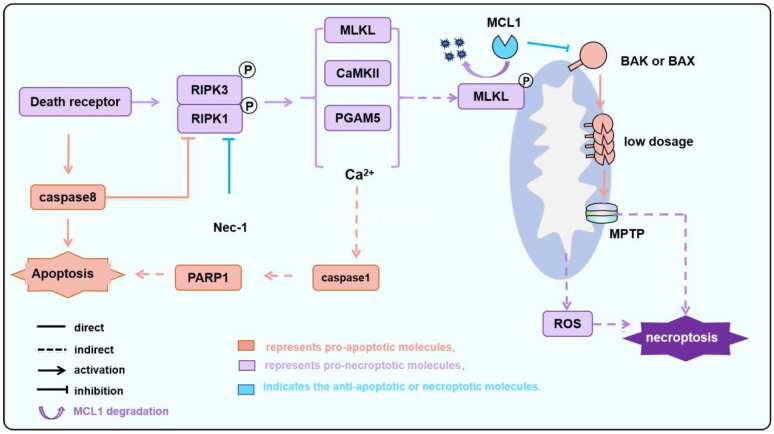
The MPTP-dependent necroptosis during IR is regulated by BAK/BAX. During IR, particularly following reperfusion, the activation of death receptor signaling leads to the activation of RIPK1/RIPK3. Subsequently, CAMKII and PGAM5, among others, induce the opening of pores formed by the F_1_-F_0_ ATP synthase and/or CypD, which is known as MPTP. Additionally, MLKL is phosphorylated and translocated to the mitochondria inducing MCL1 degradation and releasing BAK/BAX. Monomeric or a small amount of oligomerized BAK/BAX then triggers MOMP, altering the permeability of the mitochondrial outer membrane. This process facilitates or amplifies the MPTP-induced necroptosis.

In fact, apoptosis (particularly mitochondrial apoptosis) is a significant contributor to the mechanism of myocardial cell death during AMI [64,65,66,67,68,69,70]. In contrast, the MPTP-driven pathway is operational in both AMI and IR, with necroptosis emerging as a notable manifestation of cell death in this context [71,72,73]. These two types of myocardial cell death, usually induced by ischemia or hypoxia during IR, are both closely regulated by BCL2 family proteins [74] (Figure 2 and Figure 3).

In addition to causing AKT deactivation and JNK activation (see below), AMI and IR initiate cell death through two major molecules (Figure 2): rapid calcium overload and/or ROS. Mitochondria serve as one of the primary sources of ROS. Under conditions of ischemia or reperfusion injury, mitochondria generate substantial amounts of ROS, triggering various modes of cell death, including apoptosis, necroptosis, and mitophagy [75,76]. While the destructive effects of apoptosis and necroptosis on cardiomyocytes during AMI and IR are well-established and dominate the process, there remains controversy regarding the destructive or protective role of mitophagy [77,78,79]. Mechanistically, oxygen reduction during MI results in the suppression of mitochondrial oxidative phosphorylation, leading to the transformation of aerobic metabolism to anaerobic metabolism, therefore causing a decrease in intracellular pH. Due to the exchange of Na^+^-H^+^ and Na^+^-Ca^2+^, the influx of sodium and calcium into the cells is increased, resulting in an increase in acidity and intracellular Ca^2+^ levels. The rapid increase in intracellular Ca^2+^ leads to MPTP opening [80]. If the blood supply is not restored in time after ischemia, low levels of ATP and high levels of Ca^2+^ will eventually lead to MPTP-driven and MOMP-dependent apoptosis. Although reperfusion after MI is the optimal approach to prevent apoptosis of myocardial cells following ischemia [80], reperfusion therapy may also cause tissue damage. Reperfusion often leads to the excessive generation of ROS due to the sudden restoration of oxygen supply and the damaged electron transport chain during hypoxia. While the mitochondrial metabolite succinate accumulates during ischemia, upon reperfusion and restoration of oxygen, succinate is rapidly oxidized by succinate dehydrogenase (SDH), generating superoxide [80,81,82], and again triggering MPTP-driven cell death and increasing the disruption of myocardial structure and function [83], defined as reperfusion injury [84].

### 3.2. Myocardial Cell Apoptosis and Necroptosis Are Both Regulated by BCL2 Family Proteins

#### 3.2.1. Recent Advances on Structural Changes in BAX and BAK During MOMP

The mitochondrial pathway is controlled by the BCL2 protein family proteins [29,30,31,85]. Following the activation of BAK and BAX, structural changes represent a crucial step in inducing apoptosis. Studies have shown that BAK and BAX bind to the BH3 domain of BH3-only family members and subsequently oligomerize. BAX activation is reported to initiate at the N-terminus [86,87], causing the release of its α9 helix from the hydrophobic BH3-binding groove. This conformational change facilitates the translocation of BAX from the cytosol to mitochondria, followed by the binding of activator molecules (such as BH3-only molecules) to the BH3-binding groove [88]. Upon activation, the α9 helix of BAX inserts into the anchored mitochondria, and its α2–5 region forms a pocket structure capable of binding to the BH3 domain of another BAX molecule to form a dimer [89,90]. Continued polymerization of dimers converts BAX into higher-order oligomers and initiates MOMP [67,91,92,93]. Because BAX is usually cytosolic with the α9 domain occupying the canonical BH3 domain binding groove, while BAK α9 has a different feature, BAK activation is initiated by the binding of BH3-only proteins to the BH3 domain binding groove [32,94,95]. The dimerized/oligomerized BAK permeabilizes mitochondria, also involving a symmetry dimerization of the α2-α5 core. While the high-order oligomer structure of the BAX with its C-terminal transmembrane α9 domain has been solved [67] and BAK will likely have a similar oligomerization fold, it remains unclear how BAK/BAX permeabilizes the mitochondrial outer membrane. The C-terminal helices α6, α7, and α8 extend from the membrane-anchored core and can swing freely within the membrane, while these helixes can permeabilize the mitochondrial outer membrane without the other domains [96]. It is possible that during or after BAK (or BAX) structural arrangement, selective binding of BAK (or BAX) α5 and α6 recruits cardiolipin and causes MOMP. Indeed, lipids and BAK dimers rearrange to bury exposed hydrophobic surfaces, forming “pores” [97]. BAK and BAX are activated by certain BH3-only proteins [98,99,100], while BAK undergoes autoactivation at high concentrations [101]. Anti-apoptotic proteins exert inhibitory effects on activated BAK/BAX [102]. Additionally, BCL2 family proteins exhibit dynamic interactions and tendencies [103,104,105], maintaining the balance of apoptosis within cells.

#### 3.2.2. MOMP-Dependent Apoptosis in the Process of AMI and IR

The mitochondrial apoptotic pathway serves as a crucial factor during AMI and IR [71,106]. The pro-apoptotic proteins PUMA, BAX, and NOXA, as well as caspase-3 activity, peaked on the first day after MI [106]. The upregulation of PUMA, BAX, and NOXA is primarily triggered by severe oxidative stress and DNA damage during IR. Transcriptionally, PUMA and BAX are potently induced by p53 after DNA damage, while NOXA is induced by hypoxia-inducible factor 1 (HIF-1) under hypoxic conditions [107]. Mitochondrial damage, which is associated with hypoxia, also leads to a decrease in BCL2, as well as the opening of the MPTP or MOMP mediated by BAK and BAX [108,109]. Surprisingly, in an ischemic and hypoxic environment, blocking the electron transport chain results in elevated levels of BCL2, leading to a decreased sensitivity of the MOMP to apoptotic signals [27,110,111].

#### 3.2.3. MPTP-Driven Necroptosis Is Also Regulated by BCL2 Family Proteins During IR

**Figure 4:**
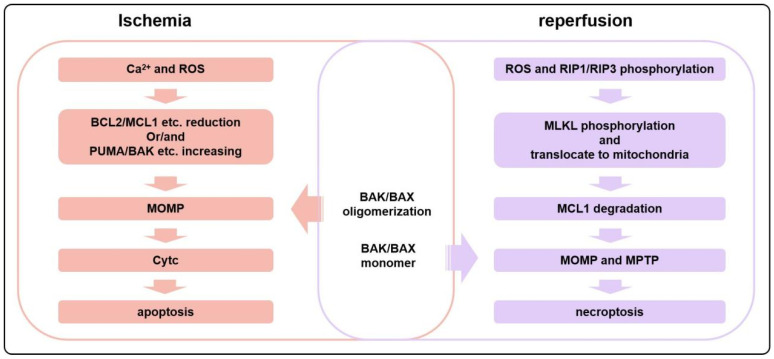
During the initial stages of ischemia, cardiomyocytes tend to undergo apoptosis more frequently, whereas necroptosis is more prevalent during reperfusion. In ischemia, an influx of Ca^2+^ and the production of a large amount of ROS induce a reduction in the amount or activities of anti-apoptotic proteins such as BCL2 or MCL1 and an increase in pro-apoptotic proteins, leading to apoptosis. However, during reperfusion, the activation of the RIP1/RIP3 pathway induces the phosphorylation and translocation of MLKL to mitochondria, resulting in the degradation of MCL1. This triggers the opening of the MPTP, as well as the BAK/BAX-dependent MOMP, ultimately leading to necroptosis.

Canonical necroptosis and MPTP-driven regulated necroptosis are regulated by different molecular mechanisms (Figure 1 and Figure 3). The former is primarily initiated by death receptor signaling or virus RNA, leading to RIPK1 or ZBP1-mediated RIPK3 phosphorylation and MLKL phosphorylation. The activated p-MLKL translocates to the plasma membrane, forming pores, resulting in cytolysis and a robust inflammatory response [26]. In contrast, MPTP-driven regulated necroptosis is defined by MIMP following persistent MPTP opening. Notably, the necroptotic pathway converges with the intrinsic apoptotic pathway at BAK/BAX in this situation. In particular, in the MPTP-driven necroptosis, activated MLKL translocates to mitochondria, promotes the degradation of the anti-apoptotic MCL1, and thereby releases sequestered BAK/BAX. The liberated BAK and/or BAX (potentially as monomers or low-order oligomers in contrast to high oligomers formed during MOMP, Figure 4) subsequently facilitate MPTP opening. In this context, BAK and BAX act as critical integrators, linking upstream death signals (including those from necroptosis) to downstream mitochondrial collapse, thereby forming a positive feedback loop that amplifies cellular demise [40,111,112]. During IR, the expression of inflammatory factors in cardiomyocytes, such as TNFα, increases, leading to the activation of inflammatory pathways, a significant increase in RIPK1/RIPK3 phosphorylation levels, and sustained activation of MLKL (Figure 3) [111]. Some other studies also suggest that MPTP opening depends on BAK/BAX monomers without oligomerization. For instance, ionomycin induces MPTP-driven necroptosis, characterized by decreased mitochondrial oxygen consumption, dysfunction, and swelling. However, these phenomena disappear after BAK/BAX double knockout; however, ionomycin-induced BAK/BAX-mediated and MPTP-driven necroptosis does not alter the conformation of BAK or BAX. Restoring the expression of the BAX mutant that is unable to oligomerize in BAK/BAX double-knockout cells can recapitulate MPTP-driven cell death but not MOMP-dependent apoptosis, showing the involvement of BAX monomer but not high oligomers in MPTP-driven necroptosis [113]. This involvement of BAX/BAK monomers but not high oligomers in MPTP-driven necroptosis might be explained by the fact that C-terminal helixes of BAK/BAX can permeabilize the mitochondrial outer membrane [96]. Furthermore, Karch et al. suggest that MPTP-driven necroptosis requires a small amount of BAX oligomerization, while MPTP-driven regulated necrosis only requires BAX monomers [40].

Some other mechanisms have also been reported to be involved in MPTP-driven cell death during IRI. For instance, the overexpression of TNFα leads to the activation of the RIPK3/MLKL pathway and disrupts the plasma membrane, while RIPK3 phosphorylates and activates CaMKII simultaneously, inducing mitochondrial calcium overload and promoting the opening of MPTP. Furthermore, RIPK3 stabilizes CypD via PGAM5, further enhancing MPTP opening (Figure 3). The sustained opening of MPTP results in the collapse of the mitochondrial membrane potential, the depletion of ATP, and the production of ROS, triggering necroptotic cell death [113] (Figure 3).

#### 3.2.4. Inhibiting MPTP-Driven Cell Death to Protect Myocardial Cell Death

Inhibiting MPTP-driven necroptosis is another crucial approach to myocardial cell protection [66,70]. When MPTP is inhibited by knocking out CypD, the damage caused by myocardial IR is significantly reduced, as evidenced by a certain degree of recovery in cardiac function and a decrease in infarction size [35]. In a separate study, knocking out CypD or administering a CypD inhibitor cyclosporine A to IR mice significantly reduced both the infarction area and the risk zone in the mouse heart [114]. The persistent opening of MPTP predominantly leads to cell death through necroptosis, but it also induces other forms of cell death, such as apoptosis, pyroptosis, and mitophagy. Because the occurrence of other forms of cell death can feed back to stimulate the continued opening of MPTP, inhibiting MPTP also mitigates myocardial damage by reducing other forms of cell death induced by the persistent opening of MPTP after myocardial IR.

In summary, both apoptosis and MTPT-driven necroptosis during AMI and IR are regulated by BCL2 family proteins. Although multiple forms of cell death, including apoptosis and MPTP-driven necroptosis, occur during AMI and IR, the timeline differs between various types of cell death. The occurrence of BAK/BAX-dependent MOMP promotes MPTP-driven necroptosis, but when a large amount of BAK/BAX oligomerizes, the cell is more likely to undergo apoptosis rather than necroptosis. During I/R, MOMP-induced apoptosis occurs early, whereas MPTP-driven necroptosis predominates during later reperfusion phases and may persist for extended periods, as observed in murine LAD models (e.g., up to 12 weeks post-MI) [70,72]. Critically, the prolonged activation of MPTP-driven necroptosis drives adverse myocardial remodeling, infarct expansion, and arrhythmias. Multiple pathological factors induce MOMP and MPTP, which are interconnected and trigger myocardial apoptosis and necroptosis, ultimately leading to mitochondrial dysfunction, swelling, myocardial hypertrophy, and myocardial remodeling.

### 3.3. Multiple Upstream Pathways Directly or Indirectly Regulate BCL2 Protein-Mediated Myocardial Cell Apoptosis and Necroptosis

#### 3.3.1. Multiple Upstream Pathways Regulate BCL2 Phosphorylation to Regulate Myocardial Cell Death

Multiple pathways have been reported to regulate BCL2 phosphorylation and thus regulate myocardial cell apoptosis and necroptosis (Table 1). BCL2, which is the first identified anti-apoptotic BCL2 family gene [115,116,117], has been shown to inhibit MOMP and subsequent cell death through its direct inhibitory effects on pro-apoptotic BAX, BIM, PUMA, NOXA, truncated BID (tBID), and, to a lesser extent, BAK [118,119,120,121]. In addition to regulating apoptosis, BCL2 has been shown to directly interact with ANT or VDAC proteins to regulate MPTP opening [122,123]. Different from the anti-apoptotic protein BCLX_L_, BCL2 has a long loop domain between helixes a1 and a2, which is necessary for BCL2 phosphorylations during mitosis or after induction by multiple reagents [124,125]. BCL2 phosphorylations have been shown to affect its anti-apoptotic function, although the effect is controversial [126,127,128].

**Figure 5:**
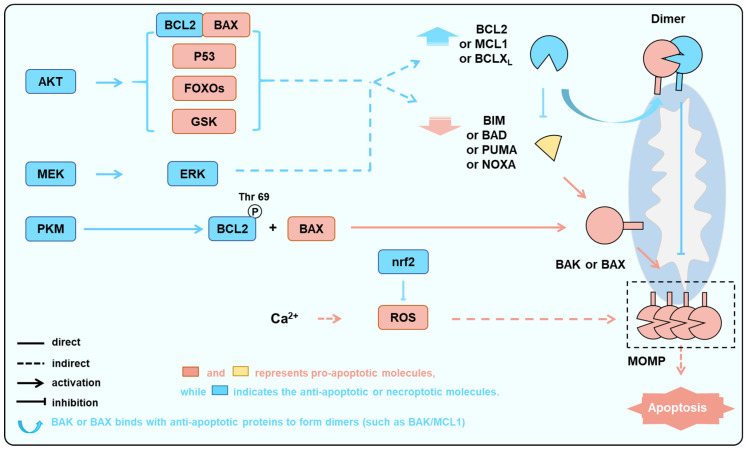
Multiple pathways aim to protect cardiomyocytes by suppressing the activation of BAK/BAX as the downstream node. In the condition of ischemia and hypoxia, the activation of kinase pathways such as AKT, ERK, and PKM increases the levels or activities of anti-apoptotic proteins and decreases the levels or activities of pro-apoptotic proteins, while the activation of the nrf2 pathway can inhibit excessive ROS-induced cellular damage. These processes ultimately aim to reduce the activation of BAK/BAX, thereby decreasing the occurrence of apoptosis.

During IR, the phosphorylation level of BCL2 is significantly elevated. Similar to its effect in cancer cells, the phosphorylation effect of BCL2 induced by different kinases and the various phosphorylation sites can lead to completely opposing apoptotic outcomes (Figure 4) [129]. In some other studies, BCL2 phosphorylation exhibits an anti-apoptotic effect. In healthy adult cardiomyocytes, the expression level of PKM2 is relatively low. However, under acute or chronic myocardial ischemia, the level of PKM2 significantly increases. The overexpression of PKM2 or the facilitation of glutathionylation at the Cys424 site of PKM2 markedly diminishes the oxidative stress and apoptosis triggered by ischemia and hypoxia (Figure 5). Additionally, it enhances cardiomyocyte proliferation, improves myocardial function, and ultimately achieves cardiac protection under I/R conditions [130]. The HSP90α1 subunit induces the exposure of the 389–405 peptide segment of PKM2 and directly binds to BCL2, thereby promoting the phosphorylation of Thr69 in BCL2. This process will reduce the degradation of BCL2 by E3 ubiquitin ligase and enhance its anti-apoptotic activity [131,132,133]. Because phosphorylated BCL2 shows increased affinity to BAK, the increase in the anti-apoptotic effect after BCL2 phosphorylation might be due to its increased affinities to BAK [118].

Some other studies indicated that BCL2 phosphorylations cause its inactivation. Kinases, such as JNK, which induce BCL2 inactivation through phosphorylation and induce apoptosis (Figure 2), leading to myocardial cell damage and apoptosis during ischemia and oxidative stress [134,135]. The JNK/c-JUN-AP-1 and JNK/NF-κB pathways in H9C2 cells are significantly activated with hypoxia. AMPK inhibition exacerbates this abnormal activation, whereas AMPK activation mitigates the abnormal activation of these pathways, ultimately reducing hypoxia/reoxygenation-mediated apoptosis. Activation of JNK significantly increases the levels of p-BCL2ser70 and p-BCL2ser87 in H9C2 cells [136,137]. Phosphorylation of BCL2 reduces its affinity for BAX, leading to its dissociation and subsequent induction of apoptosis [131,137]. In a separate study, phosphorylations at T60, S70, and S87 of BCL2 also lead to decreased anti-apoptotic activity [138].

While different conclusions were obtained regarding whether BCL2 phosphorylation anti- or pro-cell death, some of the mechanisms might explain the different outcomes: First, BCL2 phosphorylation has been shown to increase its binding to BAK [118]. Second, BCL2 phosphorylation might affect its affinities to the BH3-only proteins such as BIM, PUMA, and tBID. Third, BCL2 phosphorylation might affect its binding to some other proteins such as ANT or VDAC to regulate other types of cell death. Therefore, under different situations, the effect of BCL2 phosphorylation on myocardial cell death varies.

#### 3.3.2. Multiple Pathways Directly or Indirectly Regulate Expression Levels or Activities of BCL2 Family Proteins

Apart from regulating the phosphorylation of BCL2, multiple pathways modulate the levels of BCL2 family proteins to control cardiomyocyte death during myocardial IR [139,140]. For example, not only are BAX and caspase-3 upregulated, but BCL2 is also downregulated during IR [141]. Multiple signaling cascades have been identified to modulate cardiomyocyte apoptosis in the context of IR via the BCL2 family, including the PI3K/AKT pathway and MEK1-ERK1/2 pathway, the nrf2/HO-1 pathway, and

First, the PI3K/AKT pathway is a crucial regulator of protein synthesis and is closely associated with mitochondrial intracellular oxidation [142] (Figure 2). Research has shown that the PI3K, which is composed of the regulatory subunit p85 and the catalytic subunit p110, forms a heterodimer that plays a pivotal role in cellular signaling. Upon binding to growth factor receptors such as EGFR, PI3K triggers structural changes in the AKT protein, leading to its phosphorylation and activation. The phosphorylated AKT acts as a switch, either activating or inhibiting a range of downstream substrates [143,144], ultimately influencing cell survival, growth, and metabolism (Figure 5).

AKT regulates apoptosis by regulating BCL2 family proteins through multiple modes (Figure 2). Firstly, BAD is directly phosphorylated by AKT at Ser136 in mice (Ser97 in humans), and then bound to 14-3-3 protein, which will promote BAD dissociation from anti-apoptotic partners such as BCLX_L_ [145,146]. Secondly, AKT mediates the phosphorylation of FOXO3a, resulting in the downregulation of BIM and PUMA expression [147,148]. In addition, AKT inhibits the activity of GSK3b, which in turn induces the recognition and degradation of MCL1 by E3 ubiquitin ligases, thereby increasing the levels of anti-apoptotic proteins [149]. Previous studies have indicated that p-mTOR and p-AKT are markedly reduced in rat cardiomyocytes during IR, which will in turn affect the BCL2 family proteins and regulate apoptosis [143]. Conversely, caspase-3 and BAX expression are significantly elevated, while BCL2 expression is significantly reduced in cardiomyocytes [144]. Similarly, the neuroprotective effects of hesperidin in cerebral IR are achieved by upregulating the PI3K/AKT signaling pathway and downregulating the BAX/BCL2 ratio [150]. In the isolated rat heart, sevoflurane treatment induces the activation of the AKT signaling pathway and decreases the BAX/BCL2 ratio, reducing myocardial reperfusion injury [51].

Second, PTEN is involved in regulating the PI3K/AKT pathway in IR. PTEN is a downstream key regulator of the PI3K/AKT pathway in IR, which dephosphorylates PIP3 to PIP2, thereby suppressing PI3K/AKT. Feng et al. found that inhibiting PTEN significantly activates the PI3K/AKT/ERK pathway, exerting a pro-apoptotic influence that helps safeguard the cardiomyocytes against the deleterious effects of IR [151,152]. Increased levels of PTEN can inhibit the function of the PI3K/AKT pathway, potentially leading to cardiomyocyte apoptosis during IR [153]. Li et al. found that compared with sham-operated animals, the levels of PTEN and BAX in myocardial cells were significantly increased in the IR group, while the levels of p-AKT and BCL2 were markedly decreased [154].

Third, during MI/IR, ERK1/2 is an important protein kinase that regulates cell apoptosis in reperfusion injury [155]. Previous studies have shown that MEK-induced activation of ERK not only enhances the expression of BCL2 and BCLX_L_ but also promotes the degradation of pro-apoptotic proteins such as BIM and BAD [156,157] (Figure 5). After inhibiting the ERK1/2 pathway, the BAX/BCL2 ratio increases in myocardial cells, leading to increased cell apoptosis. Sun et al. found that activating ERK by expressing CaMEK significantly improved mitochondrial membrane potential and cell viability in HO-mediated myocardial cells, reducing myocardial cell apoptosis [158,159].

Fourth, NRF2 has a protective effect on oxidative stress response after MI, thereby reducing myocardial cell apoptosis and improving MI-induced cardiac remodeling and heart failure [160]. NRF2/HO-1 mainly maintains cellular balance and reduces oxidative stress damage. After successfully establishing the myocardial infarction model in mice for 10 days, Nrf2−/− mice showed symptoms such as myocardial hypertrophy and left ventricular dilation, rapidly progressing to heart failure, with a higher mortality rate (50% vs. 86%) compared to wild-type mice [161]. Drugs such as baicalin [162], hirudin [163], aminophenyl sulfone [164], and rosuvastatin combined with carvedilol [165] can reduce myocardial cell apoptosis after MI. The primary mechanism underlying the efficacy of these drugs involves regulating the Nrf2/HO-1 pathway, which helps to reduce ROS content in MI mice and promote SOD expression, thereby protecting myocardial cells from oxidative stress damage. Additionally, pretreatment with HO-1 or HO-1 activator A in a murine model of myocardial infarction following LAD ligation significantly reduced infarct size and improved cardiac function after 30 days [166,167]. All studies indicate that regulating Nrf2/HO-1 is a crucial approach to reducing oxidative damage during the development of I/R.

### 3.4. Inhibiting BAX/BAK-Regulated Cell Death Function Is Crucial for Achieving Cardiac Function Protection

BAX/BAK-regulated MOMP or MPTP is a key downstream node in the regulation of apoptosis and necroptosis by the BCL2 family. Targeting the homeostasis of BCL2 family proteins is an important intervention target during AMI; that is to say, cardiomyocyte apoptosis can be reduced by upregulating anti-apoptotic proteins or inhibiting pro-apoptotic proteins, which provides a potential strategy for cardioprotection. One strategy is to target BAX/BAK-mediated MPTP [168]. During the later stages of IR, inflammatory factors such as TNF-α activate the canonical necroptosis pathway involving RIPK1/RIPK3/MLKL. This activation suppresses MCL1 function, leading to the release of BAK or BAX monomers and triggering BAK/BAX-mediated necroptosis. This process disrupts the integrity of cellular or organellar membranes, thereby compromising permeability homeostasis. The subsequent massive release of intracellular contents induces a robust inflammatory response, which in turn causes secondary damage to the surrounding myocardial tissue (Figure 3).

Since BAX/BAK are essential executive BCL2 family proteins, studies have been performed to inhibit BAX/BAK to achieve cardiac protection. Karch et al. found that cardiomyocyte-specific silencing of BAX reduced infarct size in BAK−/− mice after IR injury (C57BL/6 mice, 30 min ischemia/24 h reperfusion), an effect indicating attenuated apoptosis and suggesting that the combined loss of BAX and BAK is required for maximal protection, likely by abolishing both MOMP and MPTP [40]. It has been reported that overexpression of BCL2 [8,11,169] or BCLX_L_-derived peptides [170] in transgenic mice has a preventive effect on myocardial ischemia–reperfusion. Similarly, in a mouse model of myocardial I/R injury (C57BL/6 mixed background, 20 min ischemia/120 min reperfusion), the LDH release and infarct size of PUMA−/− mice were both reduced to half of those of WT mice and PUMA heterozygous mice. The representative cardiac function index of left ventricular end-diastolic pressure (LVDP) recovered significantly (15% vs. 73%), and the animal survival rate increased from 20% to 90% [171,172]. In Des−/− mice, mitochondrial swelling and malformation were observed in cardiomyocytes, and the hearts exhibited various symptoms of cardiomyopathy, such as myocardial hypertrophy, calcification, myocardial fibrosis, and decreased heart function. However, the increased expression of anti-apoptotic BCL2 significantly alleviated the adverse conditions caused by Des deficiency [173]. Additionally, it has been reported that Neurotropin-3 protects the heart from ischemia–reperfusion injury by promoting BIM (BAK/BAX agonists) degradation through increasing the levels of p-ERK [174]. Inhibition of the caspase activities downstream of BAK/BAX-dependent apoptosis also provides some effects. In rat models of myocardial IRI (e.g., Sprague Dawley rats subjected to 30 min ischemia/24 h reperfusion), the administration of the broad-spectrum caspase inhibitor zVAD.fmk reduced infarct size by approximately 40–50% compared to vehicle controls. This effect was observed even when the inhibitor was administered after the onset of ischemia. However, the effect of zVAD.fmk failed to reduce infarct size in mouse models under similar conditions [175,176,177,178]. A study in C57BL/6J mice (30 min ischemia/24 h reperfusion) demonstrated that the XIAP-mimetic peptide PTD-BIR3/RING, which also acts downstream of MOMP, even when administered during reperfusion, reduced infarct size from 41–44% to 23–28% of the area at risk [178]. The deletion of caspase delays the occurrence of cell death, but it cannot avoid the ultimate outcome of cell death. For instance, Caspase3 and Caspase7 double knockout mice failed to demonstrate cardiac protection during RI/MI [179]. Furthermore, when mitochondria are induced by Ca^2+^ to exhibit the MPTP phenomenon, it triggers apoptosis. However, upon the double knockout of BAK and BAX, MPTP in MEF cells is reduced, resulting in the inhibition of apoptosis and necroptosis [180]. In summary, targeting BAK/BAX represents a potential strategy for achieving myocardial protection in IR conditions.

### 3.5. Effective Protection Against Cell Death by Inhibiting BAX/BAK with Small Molecules

**Figure 6:**
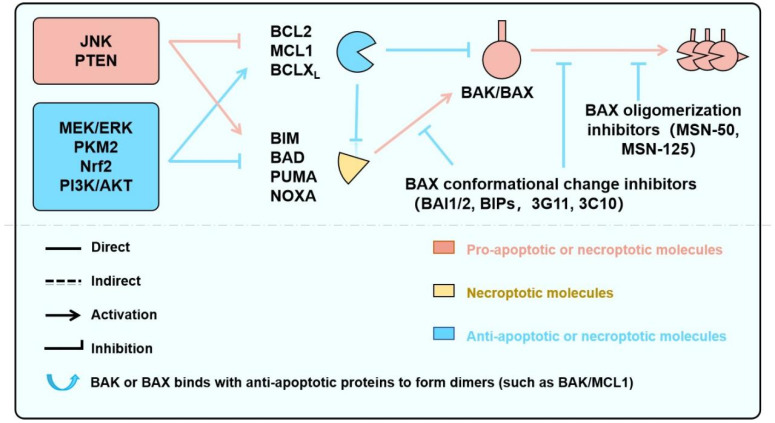
Signaling Pathways Regulating BAK/BAX-Dependent Apoptosis and Necroptosis, and Targeted Pharmacological Strategies. This schematic illustrates key upstream signaling pathways—including JNK, MEK/ERK, and PI3K/AKT—that converge on the BCL2 protein family to regulate mitochondrial outer membrane permeabilization (MOMP) and cell death. Pro-apoptotic BH3-only proteins (e.g., BIM, PUMA, BAD) and anti-apoptotic members (e.g., BCL2, MCL1, BCLX_L_) interact dynamically to control the activation of the effector proteins BAK and BAX. Their activation leads to apoptosis or, under certain conditions, necroptosis. Small-molecule screening strategies—such as BH3–groove binders, α9 helix mobilization blockers, and lipid–BAK interface disruptors—offer potential to modulate these interactions. Tool compounds targeting these nodes are primarily studied in Cardiovascular disease, neurological and cancer models.

A few small molecules have been developed to inhibit BAK/BAX and are under preclinical studies (Figure 6). Niu et al. found that MSN-50 and MSN125 could inhibit tBid-mediated activation of BAX and/or BAK in a concentration-dependent manner [181,182]. While actinomycin D (ActD) or staurosporine (STS) induces cell apoptosis in a BAK/BAX-dependent manner, MSN-50 and MSN125 significantly inhibited apoptosis of HCT116 cells induced by ActD or STS. ActD activates BAX by inducing a conformational change in BAX, while MSN-125 disrupts the proper formation of BAX dimers, thereby preventing the assembly of functional oligomers and inhibiting MOMP. MSN-125 protected cultured primary embryonic mouse cortical neurons from excitotoxicity induced by glutamate and reduced the death rate of a large number of neuronal cells. Unlike other neuroprotective agents, using MSN-125 to inhibit BAX/BAK oligomerization after excitotoxic injury still protected neurons [182].

As we discussed, the induction of cell death by BAK/BAX relies on conformational changes that occur upon their activation. A comprehensive understanding of these conformational changes holds promise for the development of BAK/BAX inhibitors. However, the precise mechanisms underlying the conformational changes following BAX/BAK activation remain incompletely elucidated, thereby hindering progress in developing effective BAK/BAX inhibitors. Using tBID-mediated BAX activation as a model, Thomas et al. found [183] that the imidazole compound BAI1 effectively inhibited BAX-mediated MOMP; this compound inhibited the induction of BAX conformational changes by tBID or BIM, including mobilization of the BH3 domain and C-terminal helix α9 from the hydrophobic core. BAI1 effectively inhibited TNFα and ceramide-induced activation of BAX and BAK, thereby reducing apoptosis in MEF cells. Some researchers have targeted BAX to develop short peptide inhibitors, which significantly improve the progression of type I diabetes [184] or pulmonary fibrosis [185], but research on BAX/BAK inhibitors for IR has not yet been conducted. Currently, there are still relatively few studies on BAX/BAK inhibitors, and the conformational changes following BAX/BAK activation are not yet clear, requiring further research on the mechanism of BAX/BAK activation.

In the pathophysiology of IR, the BCL2 protein family is a master regulator of cell death, wherein the pro-apoptotic effectors BAK/BAX stand out as key molecules that regulate both MOMP-dependent apoptosis and MPTP-driven necroptosis, ultimately deciding the survival fate of cardiomyocytes.

## 4. Discussion and Prospect

In summary, during the development of ischemic cardiomyopathy, the activation of BAK/BAX by multiple pathways plays an essential role in cell apoptosis and necroptosis [68,69,186], ultimately resulting in poor prognosis and increased mortality in patients with IR. Direct targeting of BAK/BAX to inhibit cardiomyocyte apoptosis and necroptosis is an effective approach to improve the poor prognosis of myocardial infarction. Accumulating preclinical evidence underscores the therapeutic promise of targeting the mitochondrial cell death pathway in myocardial IR. Strategies such as enhancing BCL2 or BCLX_L_ activity, promoting degradation of the agonist BIM, or inactivating caspases have been shown to mitigate damage, with the most compelling evidence coming from BAK/BAX knockout mice, which exhibit markedly improved outcomes. Although the exact activation mechanism is not fully elucidated and pharmacological inhibitors remain exploratory, the principle of BAK/BAX as a final common effector suggests its therapeutic potential likely extends beyond AMI/IR. This implies relevance for acute myocarditis, global cardiac arrest, and the controlled IR of cardiac surgery, conditions sharing features of mitochondrial dysfunction. To fully realize this broad potential, a deeper mechanistic understanding is crucial. Future efforts must decipher the relative significance of BAK/BAX across cell death subroutines and elucidate the structural biology of its activation and pore formation, which will be essential for developing effective and targeted therapeutics. Currently, there are only a few studies on BAX/BAK inhibitors and several studies on the correlation between BAK/BAX knockout and IR, while no drug discovery research has been conducted on BAX/BAK inhibitors for the treatment of IR. It is noteworthy that systemic targeting of BAK or BAX may interfere with physiological apoptosis in normal cells, such as those in the hematopoietic and nervous systems, thereby increasing tumor risk and disrupting immune homeostasis.

Moreover, although targeting mitochondria offers novel therapeutic perspectives for myocardial ischemia–reperfusion injury, its clinical translation faces substantial challenges. The clinical trials of MPTP inhibitors like cyclosporine A have not achieved consistent success, primarily limited by two major obstacles: inadequate delivery efficiency and systemic toxicity. The double-membrane structure of mitochondria and the collapse of the mitochondrial membrane potential under pathological conditions significantly hinder effective drug delivery, while nonspecific distribution of these drugs can disrupt normal tissue metabolism, leading to dose-limiting toxicities. In contrast, targeting BAK/BAX demonstrates unique advantages. This strategy achieves enhanced selectivity by specifically blocking the terminal execution steps of cell death. As one of the effects of inhibiting BAK/BAX is attenuating the apoptosis, it also needs to be noted that BAK/BAX inhibition might cause certain cancers. Therefore, short-term inhibition of BAK/BAX might be a possible strategy to decrease the risk of tumorigenesis. On the other hand, its therapeutic window spans the commitment phase of cell death, providing a broader treatment opportunity. Moreover, the advent of novel delivery technologies such as nanoparticle-mediated cardiac targeting, AAV-based cardiac-specific promoters, and PROTACs technology holds promise for precise drug delivery to diseased tissues, thereby circumventing the systemic risks associated with conventional mitochondrial drugs. For example, the liver, with its abundant blood supply and inherent role as a protein synthesis factory, is highly amenable to AAV-mediated gene delivery. The use of liver-specific promoters (e.g., TBG and ALB) allows therapeutic transgene expression to be precisely restricted to hepatocytes, enabling a single injection to achieve sustained therapeutic protein production. This strategic paradigm underlies the success of approved drugs like Roctavian, Hemgenix, and Beqvez.

Therefore, conducting drug research targeting BAX/BAK for the treatment of ischemic cardiomyopathy may have broad prospects, with strategies such as nanoparticle-mediated cardiac targeting, AAV-based cardiac-specific promoters, and PROTACs technology representing promising approaches to enhance drug efficacy while minimizing clinical risks (Figure 6).

## 5. Conclusions

During the development of ischemic cardiomyopathy, the activation of BAK/BAX by multiple pathways leads to cell apoptosis and necroptosis, ultimately resulting in poor prognosis and increased mortality in patients with MI/IR. Direct targeting of BAK/BAX to inhibit cardiomyocyte apoptosis and necroptosis provides a means to limit myocardial cell apoptosis and necroptosis and therefore could potentially improve the poor prognosis of the MI/IR. Further preclinical and clinical drug studies targeting BAK/BAX for the treatment of cardiomyopathy may have broad prospects.

## Figures and Tables

**Table 1:** Multiple pathways directly or indirectly regulate BCL2 protein-mediated myocardial cell apoptosis and necroptosis.

	Model and Protocol	Role Played in IR Injury and Cardioprotection	Mechanism
JNK	① Neonatal mice cardiomyocytes or H9C2 treated with H_2_O_2_ to induce oxidative stress (a major contributor to MI injury) ② diabetic rats after ischemia/reperfusion (I/R)	Injury	Induce phosphorylation of BCL2 ser87/70, resulting in the loss of its anti-apoptotic function
PKM2	U87 or U251 cells were treated with or without H_2_O_2_	protect	Induce phosphorylation of BCL2 Thr69, reducing BCL2 protein degradation
PI3K/AKT	myocardial ischemia–reperfusion injury in rats	protect	increase BCL2/BAX ratio
PTEN	Left anterior descending arteries (LAD) of mice ligated to induce MI	Injure	increase BAX level
ERK	H_2_O_2_-induced injury in h9c2 cardiomyocytes	protect	increase BCL2/BCLX_L_ level, decrease BIM/BAD level
NRF2	Left anterior descending arteries (LAD) of mice ligated to induce MI	protect	decrease ROS and increase SOD
BCL2/BCLX_L_	a rabbit or pig model of ischemia–reperfusion injury	protect	MOMP or MPTP
BAK/BAX	a mouse model of ischemia–reperfusion injury, and knockout BAK and BAX	Injury	MOMP or MPTP
PUMA	a mouse model of ischemia–reperfusion injury and knockout PUMA	Injure	MOMP or MPTP

## Data Availability

No new data were created or analyzed in this study. Data sharing is not applicable to this article.
